# Superposition Analysis: A Method for Liver Disease Diagnosis and Prognostic Evaluation Related Gene Screening

**DOI:** 10.3390/mps9050147

**Published:** 2026-10-09

**Authors:** Xueqiang Guo, Lingling Xi, Chunbo Zhang, Zihui Wang, Qijie Xue, Jianlin Guo, Weidong Kong, Cunshuan Xu

**Affiliations:** 1 Institutes of Health Central Plain, Henan Medical University, Xinxiang 453003, China; 211004@hamu.edu.cn (X.G.); 212013@hamu.edu.cn (L.X.); 2 College of Life Science, Henan Normal University, Xinxiang 453007, China; 15238296962@139.com (C.Z.); zhwangsdhy@163.com (Z.W.); xueqijie2020@163.com (Q.X.); gjianlin@yeah.net (J.G.); 3 State Key Laboratory for Cell Differentiation Regulation, Xinxiang 453007, China

**Keywords:** rat liver disease, liver tissue gene expression profile, superposition analysis method, liver disease prediction, liver disease prognosis

## Abstract

To predict the types, stages and prognostic evaluation of liver diseases based on gene expression changes in liver tissues, the “superposition analysis method” was employed to screen mRNAs which were significant, differentially expressed, relevant, critical, and prognostically informative. Differential expression of candidate critical mRNAs in rat liver diseases was assessed using the *t*-test, and mRNAs with a *p*-value ≤ 0.01 were designated as prognostic mRNAs. The expression levels of the prognostic mRNAs were quantified by real-time PCR. The superposition analysis method was applied to calculate the weighted minimum Manhattan distance, which served as the basis for predicting the type of liver disease. 328 critical mRNAs were identified from the rat liver tissues with seven aforementioned liver diseases. Among them, six prognostic mRNAs, including *ANXA2*, *AVPR1A*, *CLEC10A*, *FABP4*, *GSTP1* and *RCN2*, exhibited *p*-values ≤ 0.01. qPCR revealed that *AVPR1A* was downregulated in rat hepatocellular carcinoma (HCC), whereas others were upregulated. The superposition analysis method demonstrated prediction accuracies of 100% for hepatitis, advanced HCC, and terminal-stage HCC; 90% for hepatic fibrosis and early-stage HCC; 80% for cirrhosis; and 50% for intermediate-stage HCC, which described herein for predicting rat liver diseases exhibits considerable reliability and feasibility.

## 1. Introduction

The liver diseases encompass hepatitis, non-alcoholic fatty liver disease, hepatic fibrosis, liver cirrhosis, and hepatocellular carcinoma (HCC) with distinct interrelatedness and a continuous progressive trajectory. For instance, chronic hepatitis and fatty liver disease can gradually trigger hepatic fibrosis; without timely intervention, fibrosis may advance to cirrhosis, which ultimately elevates the risk of HCC onset, forming a complete pathological progression cascade. This poses substantial challenges to the early screening, precise diagnosis, and prognostic evaluation of liver disorders [1]. Currently, a standardized clinical diagnosis and treatment framework has been established for liver diseases, particularly for the diagnosis and prognostic assessment of early-stage HCC, including imaging examinations, serum biomarker assays, and liver biopsy [2,3]. Alpha-fetoprotein (AFP), the most widely utilized serum biomarker for HCC, is routinely adopted in general physical examinations and clinical screenings due to its straightforward testing procedures and minimal invasiveness. Nevertheless, this standalone biomarker lacks sufficient sensitivity for identifying early liver cancer, leading to relatively high rates of missed and misdiagnoses [3]. Imaging modalities such as computed tomography (CT), ultrasonography, and magnetic resonance imaging (MRI) evaluate lesion severity by assessing liver morphology, tissue density, and secondary pathological signs. These tools exhibit strong diagnostic accuracy only for advanced cirrhosis and classic HCC lesions, yet deliver low sensitivity for mild early hepatic fibrosis and microcarcinoma foci, failing to detect incipient hepatic lesions with precision. Liver biopsy, regarded as the “gold standard” for liver disease diagnosis, enables direct visualization of pathological tissue damage. However, this invasive procedure carries clinical risks including sampling bias, local infection, and hemorrhage, while inflicting physical discomfort on patients. It is therefore unsuitable for large-population screening and long-term serial prognostic monitoring, presenting prominent limitations in routine clinical practice [2,4]. In summary, conventional diagnostic and therapeutic approaches fail to balance safety, diagnostic sensitivity, and accessibility, rendering them inefficient for achieving early detection, accurate subtyping, and refined prognostic stratification of liver diseases.

Driven by the deep integration of life sciences and information technology, high-throughput biological detection technologies are undergoing continuous iterative upgrades [5,6,7,8]. Combined with analytical frameworks including big data biology and bioinformatics, these advances have opened new avenues to break through the bottlenecks of conventional liver disease diagnosis and treatment, emerging as a cutting-edge research focus for precise disease diagnosis and prognostic evaluation. High-throughput biological testing enables high-volume, efficient, and accurate acquisition of multi-omics biological data from liver tissues, covering the genome, transcriptome, proteome, metabolome and beyond. It facilitates comprehensive exploration of microscopic molecular signatures underlying the initiation and progression of liver diseases, overcoming the limitations of traditional single-marker and single-dimensional testing, and holds promise for precisely capturing abnormal molecular signals indicative of occult early hepatic lesions. Building on this foundation, analytical methodologies from big data biology and bioinformatics support standardized processing, filtering, integration and in-depth mining of massive, complex high-throughput datasets. Such workflows efficiently eliminate redundant and invalid data and mitigate interference from measurement errors. By constructing computational models, researchers can identify distinct molecular expression patterns specific to various liver disease subtypes and pathological stages. This noninvasive, high-efficiency, scalable analytical paradigm effectively compensates for the drawbacks of traditional diagnostic and therapeutic techniques—namely insufficient diagnostic sensitivity, substantial invasiveness, and poor suitability for longitudinal dynamic monitoring. It delivers robust technical backing for the clinical implementation of precision liver medicine and boasts broad prospects for clinical translation and application.

To further refine the diagnostic and prognostic evaluation system for liver diseases and address the drawbacks of existing high-throughput data analytical approaches—including poor targeting efficiency, low data utilization rates, and limited predictive accuracy—this study innovatively establishes a big-data biological research framework termed the “superposition analysis method” tailored to high-throughput transcriptome sequencing data of liver disorders. Centered on the continuous and progressive pathological nature of liver diseases, this method follows a core logical workflow consisting of multi-factor superposition integration, feature weight assignment, and validity verification of predictive outputs. It systematically processes high-throughput biological datasets from patients at distinct pathological stages, enabling subtyping, grading diagnosis, and prognostic stratification of various liver conditions. Experimental results demonstrate that compared with conventional clinical assays and standard bioinformatic pipelines, the proposed superposition analysis model exhibits prominent strengths: cost-effectiveness, easy operability, and superior detection accuracy. The model supports streamlined early screening, precise diagnosis, and refined prognostic assessment of liver diseases, and is expected to greatly improve the accessibility of liver disease testing. Meanwhile, it expands the application ecosystem of big-data biology within precision hepatology, delivering novel analytical frameworks and technical references for mechanistic investigations, clinical diagnostic optimization, and accurate prognostic evaluation of all progressive liver diseases.

## 2. Materials and Methods

### 2.1. Acquisition of Gene Expression Profiles in Rats Liver Tissue with Liver Disease

Gene expression profiles in liver tissue derived from rats with/without liver disease were obtained from the Gene Expression Omnibus (GEO) database: https://www.ncbi.nlm.nih.gov/geo/ (accessed on 5 March 2023). The specific retrieval procedures were as follows: the keyword “hepatocellular carcinoma” was adopted for searching, with the species restricted to “*Rattus norvegicus*” and the data type set as “Series”. Only datasets containing more than three samples were selected as the source of research data.

### 2.2. Screening of Significant mRNAs in Rats Liver Tissue with Individual Liver Disease

The ratio of the mRNA signal intensity of each individual sample to that of the corresponding control sample was calculated to obtain the ratio value of the target mRNA. Genes were classified according to the following criteria: Genes with all ratio values ≥ 2 were defined as upregulated genes, while those with all ratio values ≤ 0.50 were classified as downregulated genes. For genes featuring both ratio values ≥ 2 and ratio values ≤ 0.5, the counts of ratio values falling into each category were tallied separately; the category with the larger count determined the regulatory pattern of the gene (either upregulated or downregulated).

In cases where the number of ratio values ≥ 2.0 equalled the number of ratio values ≤ 0.5, further calculations were performed as follows:

The mean value of all ratio values ≥ 2.0 was computed and divided by 2 to generate a quotient for the upregulated group.

The mean value of all ratio values ≤ 0.5 was computed and divided by 0.5 to generate a quotient for the downregulated group.

Subsequently, the two quotients were compared. If the quotient of the upregulated group (ratio ≥ 2.0) exceeded that of the downregulated group (ratio ≤ 0.5), the gene was designated as upregulated; otherwise, it was categorised as downregulated. All mRNAs exhibiting such markedly upregulated or downregulated expression profiles are collectively referred to as “significant mRNAs”.

### 2.3. Screening of Differentially Expressed mRNAs in Rats Liver Tissues with Individual Liver Disease

IBM SPSS Statistics 26.0 software was utilised to conduct independent-samples t-tests on the expression levels of each mRNA for all liver disease groups versus the control group. mRNAs with a *p*-value < 0.05 were defined as “differentially expressed mRNAs”.

### 2.4. Screening of Liver Disease-Associated mRNAs in Rats with Individual Liver Disease

The “significant mRNAs” obtained from Materials and Methods 2 and the “differentially expressed mRNAs” identified in Materials and Methods 3 were intersected to screen mRNAs satisfying both screening criteria. Such mRNAs that were both statistically significant and differentially expressed were defined as liver disease-associated mRNAs for individual samples.

Furthermore, the liver disease-associated mRNAs derived from all samples of the same liver disease subtype were integrated, and mRNAs shared across every single sample were designated as “liver disease-associated mRNAs” for that disease. By applying this identical procedure sequentially, “liver disease-associated mRNA” sets were obtained for all seven categories of liver disease.

### 2.5. Screening of Liver Disease-Critical mRNAs in Rats with Multiple Liver Diseases

The “liver disease-associated mRNAs” identified for the seven liver disease types were further intersected. mRNAs shared by them were defined as “liver disease-critical mRNAs”.

### 2.6. Screening of Liver Disease-Prognostic mRNAs in Rats with Multiple Liver Diseases

The “liver disease-critical mRNAs” derived from the aforementioned rat tissue samples were subjected to differential expression analysis separately. Those mRNAs with a *p*-value < 0.01 were defined as “liver disease-prognostic mRNAs”. Subsequently, hierarchical clustering analysis [9], together with functional annotation and signalling pathway enrichment analysis, were performed on the expression profiles of the liver disease-critical and -prognostic mRNAs via the Metascape platform (v3.5.20250701) [10].

### 2.7. Calculation of Mean Ratio Values for Liver Disease-Prognostic mRNAs

For each “liver disease-prognostic mRNA”, all ratio values obtained from rats liver tissue samples with liver disease were collected, and then mean was calculated. This mean value was used as the expression characteristic value of the “liver disease-prognostic mRNA” in the samples.

### 2.8. Establishment of the Superposition Analysis Method and Liver Disease Prediction

The “superposition analysis method” proposed in this study incorporates four components: the mean ratio values of prognostic mRNAs, gene expression template values for liver diseases, calculation of weighted Manhattan distance, and liver disease prediction.

When calculating the mean ratio values of “liver disease-prognostic mRNAs”, the values of each liver disease samples were determined in accordance with Materials and Methods 8. For the construction of expression template values, the median of the mean ratio values of all “liver disease-prognostic mRNAs” within the same liver disease samples was adopted as the gene expression template value for that disease.

For weighted Manhattan distance calculation, the mean ratio values of “liver disease-prognostic mRNAs” across the described samples in Materials and Methods 8 were used. The coefficient of variation (CV), defined as standard deviation divided by the mean, was computed for “liver disease-prognostic mRNA”. The gene with the highest CV was assigned a weight of 5, the second-ranked gene a weight of 3, and all remaining genes a weight of 1.

The weighted Manhattan distance was then calculated using the formula:

$$D=\sum \begin{array}{c} 6\\ i=1 \end{array}\;w_{i}\times \left|x_{i}-t_{i}\right|$$

where *D* denotes the weighted Manhattan distance, *w_i_* represents the gene weight, *x_i_* stands for the mean ratio value of the prognostic mRNA in a given liver disease sample, and *t_i_* indicates the liver disease expression template value in a given liver disease type. In liver disease prediction, the weighted Manhattan distance between the test sample and the expression template of each liver disease type was calculated sequentially. The liver disease corresponding to the minimum distance was taken as the predicted category of the sample.

### 2.9. Evaluation of the Accuracy of Liver Disease Prediction Using the Superposition Analysis Method

The prediction accuracy of the “superposition analysis method” was calculated as the number of liver disease samples with correct predictions divided by the total number of predicted samples. A higher accuracy value indicates greater reliability of the prediction outcomes.

### 2.10. Real-Time Quantitative PCR (qPCR) Detection of Liver Disease-Prognosis mRNAs

The rat HCC cells CBRH-7919 cultured in the DMEM-High glucose medium (Gibco, USA), but rat normal hepatic cells BRL-3A in the DMEM-Low glucose medium (Gibco, Waltham, MA, USA), which containing 10% serum (Gibco, Waltham, MA, USA) and 1% penicillin/streptomycin (Gibco, Waltham, MA, USA) in a humidified incubator (37 °C, 5% CO_2_, 95% humidity), were harvested during the logarithmic growth phase. Total RNA was extracted using TRIzol reagent and reverse-transcribed into cDNA. qPCR was performed using *GAPDH* as the internal reference gene. The primer sequences are listed in Table 1 and were synthesized by Servicebio Technology Co., Ltd. (Wuhan, China).

## 3. Result

### 3.1. Expression Patterns and Functional Analysis of Rat Liver Disease-Critical mRNAs

To reduce the influence of imbalanced sample sizes among different liver disease categories on subsequent gene screening and disease prediction, no more than 10 samples were randomly selected from each liver disease type. As shown in Table 2, 59 samples were included for the identification of significant, differentially expressed, liver disease-associated, -critical, and -prognostic mRNAs, as well as for liver disease classification and staging.

**Figure 1:**
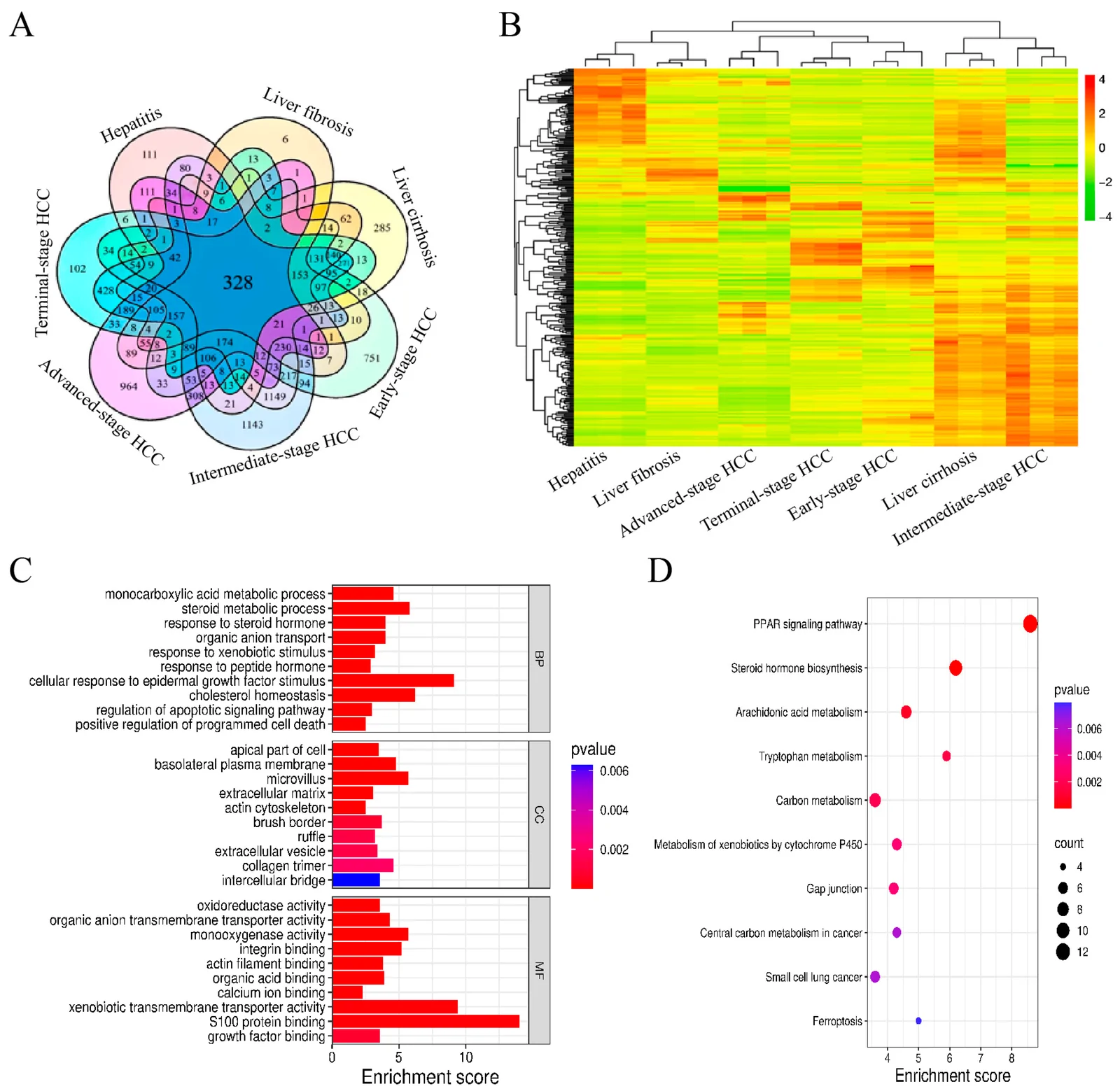
Expression patterns and functional analysis of liver disease-critical mRNAs in rat. Venn (**A**), Hierarchical clustering heatmap (**B**), GO enrichment analysis (**C**), and KEGG pathway enrichment analysis (**D**) of liver disease-critical mRNAs in rat.

High-throughput mRNA expression data from 7 hepatitis samples, 10 liver fibrosis samples, 10 liver cirrhosis samples, 10 early-stage HCC samples, 10 intermediate-stage HCC samples, 3 advanced-stage HCC samples, and 9 terminal-stage HCC samples were analyzed using the element accumulation method [11]. The numbers of significant mRNAs identified in these seven disease states were 5107, 1265, 7980, 5062, 10,213, 4717, and 3282, respectively. The corresponding numbers of differentially expressed mRNAs were 14,665, 13,593, 14,298, 14,936, 15,919, 13,441, and 13,601, respectively. The numbers of liver disease-associated mRNAs were 4431, 1265, 7453, 3754, 9747, 4585, and 3282, respectively. A total of 328 liver disease-critical mRNAs were ultimately identified across the seven liver disease states (Figure 1A). Analysis of the expression patterns of liver disease-critical mRNAs showed that the seven liver disease states exhibited distinct molecular expression profiles. Among them, hepatitis and liver fibrosis showed relatively similar expression patterns, whereas liver cirrhosis, early-stage HCC, intermediate-stage HCC, advanced-stage HCC, and terminal-stage HCC displayed distinct gene expression characteristics (Figure 1B). Gene Ontology (GO) functional annotation indicated that these critical mRNAs were mainly associated with plasma membrane components, extracellular matrix components, monocarboxylic acid metabolism, responses to xenobiotic stimuli, positive regulation of programmed cell death, oxidoreductase activity, and transmembrane transporter activity (Figure 1C). Kyoto Encyclopedia of Genes and Genomes (KEGG) pathway enrichment analysis further showed that the corresponding genes were primarily involved in steroid hormone biosynthesis, arachidonic acid metabolism, and the PPAR signaling pathway (Figure 1D).

### 3.2. Expression Patterns, Functional Analysis and qPCR Validation of Rat Liver Disease-Prognostic mRNAs

**Figure 2:**
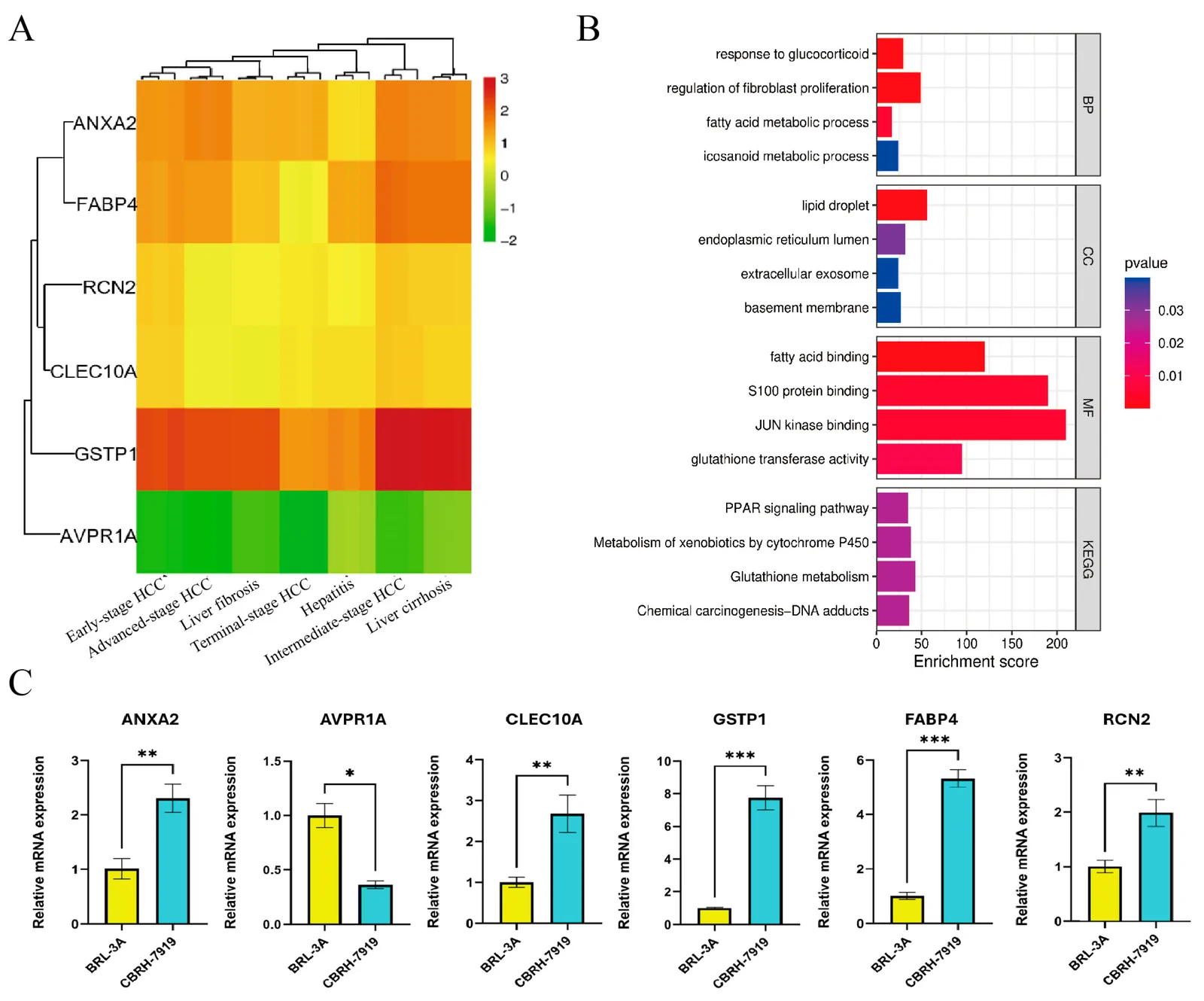
Expression patterns, functional analysis and qPCR validation of liver disease-prognostic mRNAs in rat. Hierarchical clustering heatmap (**A**), GO enrichment analysis (**B**), and qPCR validation (**C**) of liver disease-prognostic mRNAs. * *p* < 0.05, ** *p* < 0.01, *** *p* < 0.001.

Using the element accumulation method we reported previously [11], six liver disease-prognostic mRNAs with *p* ≤ 0.01 were identified from the 328 liver disease-critical mRNAs described above (Figure 1A), which including annexin A2 (*ANXA2*), arginine vasopressin receptor 1A (*AVPR1A*), C-type lectin domain containing 10A (*CLEC10A*), fatty acid-binding protein 4 (*FABP4*), glutathione S-transferase pi 1 (*GSTP1*), and reticulocalbin 2 (*RCN2*). Expression patterns analysis showed that *ANXA2* and *FABP4* displayed similar expression profiles, as did *RCN2* and *CLEC10A*. *AVPR1A* showed consistently low expression, whereas *GSTP1* was highly expressed in liver cirrhosis and intermediate-stage HCC (Figure 2A). GO indicated that they were associated with lipid droplets, the endoplasmic reticulum lumen, fatty acid binding, JUN kinase binding, S100 protein binding, glutathione transferase activity, fatty acid metabolism, response to glucocorticoids, and regulation of cell proliferation. KEGG further showed that these genes were mainly involved in the PPAR signaling pathway and glutathione metabolism (Figure 2B). qPCR demonstrated that *ANXA2*, *CLEC10A*, *FABP4*, *GSTP1*, and *RCN2* were upregulated, whereas *AVPR1A* was downregulated in rat HCC cells (CBRH-7919), which compared with normal hepatic cells (BRL-3A) (Figure 2C).

### 3.3. Concordance Between Liver Diseases Predicted by the Superposition Analysis Method and Pathological Diagnoses

Analysis performed according to the procedure described in Section 2.9 showed that the mean ratio values of the six prognosis-related mRNAs are presented in Table 3 and Supplementary Figure S1. The liver disease-specific expression signature template values and gene weights are shown in Table 4. The concordance between liver diseases predicted by the superposition analysis method and those diagnosed pathologically is summarized in Table 5.The prediction accuracy was 100% for hepatitis, advanced-stage HCC, and terminal-stage HCC; 90% for liver fibrosis and early-stage HCC; 80% for liver cirrhosis; and 50% for intermediate-stage HCC.

## 4. Discussion

Previous studies have shown that chronic liver injury may progress stepwise from hepatitis to hepatic fibrosis, cirrhosis, and eventually hepatocellular carcinoma (HCC) [12]. This process is closely associated with hepatic stellate cell activation induced by persistent inflammation, abnormal extracellular matrix deposition, immune dysregulation, metabolic alterations, and changes in gene expression [13,14]. In the present study, we developed a superposition analysis method to analyze gene expression alterations in rat liver disease samples and to predict liver disease categories and stages. Furthermore, this study extends the application of this method from liver regeneration to predicting liver disease in rats. In this method, prognosis-related mRNAs were first identified, their mean ratio values were then calculated, and liver disease-specific expression signature templates and weighted Manhattan distances were subsequently generated. The liver disease type corresponding to the smallest weighted Manhattan distance was assigned as the predicted category. By averaging multiple ratio values for the same gene, the superposition analysis method may reduce the influence of technical noise and errors derived from single measurements. In addition, this ratio-based strategy may help attenuate systematic scale differences introduced by different experimental batches, thereby improving the robustness of disease prediction [15].

Comparison of the prediction results obtained using the superposition analysis method with previously reported diagnostic models suggested that this method showed favorable performance in several disease stages. Jiang et al. [16] used machine learning algorithms, including random forest and gradient boosting machine models, to predict liver disease progression. In their study, the random forest model performed well in the hepatitis stage, whereas the gradient boosting model showed favorable performance in terminal-stage HCC, which was generally consistent with the prediction results of the present study. For liver cirrhosis and early-stage HCC, the prediction accuracy reported in their study was lower than that obtained using the present method. Wang et al. [17] proposed a radiomics-based R-score model, which achieved prediction accuracies of 87.4% and 79.1% for significant fibrosis and cirrhosis, respectively; these values were slightly lower than the accuracies of 90% and 80% obtained in the present study. Dong et al. [18] developed a computed tomography-based radiomics model for predicting Barcelona Clinic Liver Cancer (BCLC) stages. Their model achieved an accuracy of 81.8% for early-stage HCC (BCLC A), which was lower than the 90% accuracy observed in the present study, whereas its accuracy for intermediate-stage HCC (BCLC B) was 100%, which was higher than that obtained in the present study. These differences may be related to the transitional molecular features of intermediate-stage HCC. At the molecular level, intermediate-stage HCC may represent a heterogeneous transition between chronic liver disease and malignant transformation, resulting in overlapping stage boundaries and reduced classification accuracy. Nevertheless, direct comparisons among these studies should be interpreted cautiously because of differences in datasets, disease models, sample sizes, and analytical strategies. Further studies with larger sample sizes and repeated sampling validation are needed to improve the robustness and predictive accuracy of the superposition analysis method.

The six prognosis-related genes identified in this study have been previously implicated in the biological processes and molecular mechanisms underlying liver disease progression. For example, *ANXA2* has been associated with tumor invasion, progression, and poor outcomes in HCC [19]. *FABP4* has been reported to promote metabolic reprogramming and tumor progression in metabolism-associated steatotic liver disease-related HCC [20]. *GSTP1* is regulated by stress-related pathways such as the Nrf2 signaling pathway in HCC, and changes in *GSTP1* expression have been linked to tumor cell behavior [21,22]. *AVPR1A*, as a vasopressin receptor, has functional relevance in models of cirrhosis-associated portal hypertension [23]. *CLEC10A*, a receptor associated with myeloid dendritic cells, has been implicated in tumor immune regulation and immunotherapeutic strategies [24,25]. In addition, *RCN2*, an endoplasmic reticulum lumen calcium-binding protein, has been reported to be upregulated in HCC tissues and cells and to be associated with poor prognosis. Mechanistically, *RCN2* may promote HCC progression through YY1-mediated upregulation, stabilization of MYC protein, and activation of related signaling pathways [26]. These findings support the biological relevance of the prognosis-related genes identified in this study and suggest their potential value in the diagnosis, treatment, prevention, and prognostic evaluation of liver diseases. They also provide biological support for the potential applicability of the superposition analysis method reported here.

## 5. Conclusions

The superposition analysis method was employed to screen mRNAs, which were significant, differentially expressed, relevant, critical, and prognostically informative. In total, 328 key mRNAs were identified from liver tissues of rats suffering from the seven aforementioned liver diseases. Among these candidates, six prognostic mRNAs (*ANXA2*, *AVPR1A*, *CLEC10A*, *FABP4*, *GSTP1*, and *RCN2*) had *p*-values ≤ 0.01. qPCR analysis indicated that *ANXA2*, *CLEC10A*, *FABP4*, *GSTP1* and *RCN2* were upregulated, while *AVPR1A* was downregulated in the rat hepatoma cell line CBRH-7919. Gene expression profile analysis using superposition analysis achieved predictive accuracies of 100% for hepatitis, advanced HCC and terminal-stage HCC; 90% for hepatic fibrosis and early-stage HCC; 80% for cirrhosis; and 50% for intermediate-stage HCC. Compared with other predictive methods, the approach presented in this work possesses favourable reliability and feasibility for predicting liver diseases in rats.

## Figures and Tables

**Table 1:** Primer sequences of liver disease-prognostic mRNAs.

Liver Disease-Prognostic mRNA	Forward Primer (5′–3′)	Reverse Primer (5′–3′)
*AVPR1A*	ATCTGCTACCACATCTGGCG	TTATGAAAGGGACCCACGGC
*ANXA2*	GGGACTGATGAGGACTCCCT	CCTCTGCCCGTTTACCCTTT
*CLEC10A*	CTGGCATGATCCTAATTTCACCTC	GCAGATGAGTGGGTCGGAGTT
*FABP4*	AATGTGCGACGCCTTTGTG	TCTGACCGGATGACGACCAA
*GSTP1*	GGTTACCATAGATGTCTGGCTTCA	ATAAAGCCCTAAAGAGCGACCC
*RCN2*	TGACTCGGACTCTGATGGCT	CGTCAAAGTCAATCACGCGG
*GAPDH*	CTGGAGAAACCTGCCAAGTATG	GGTGGAAGAATGGGAGTTGCT

**Table 2:** Distribution of rat liver tissue samples with liver disease obtained from GEO datasets.

Liver Disease Type	Dataset of Liver Tissue from Rats with Liver Disease							
	GSE 182860	GSE 141090	GSE 44106	GSE 19507	GSE 27638	GSE 24600	GSE 157919	Total
Hepatitis	4	3						7
Liver fibrosis			20			16		36
Liver cirrhosis	8	3		9				20
Early-stage HCC			9				3	12
Intermediate-stage HCC	8				3	14		25
Advanced-stage HCC		3						3
Terminal-stage HCC			9					9

**Table 3:** Average ratio values of prognostic mRNAs for seven liver diseases.

Liver Disease State	Sample No.	Average Ratio of Liver Disease-Prognostic mRNAs					
		*AVPR1A*	*RCN2*	*ANXA2*	*CLEC10A*	*FABP4*	*GSTP1*
Hepatitis	1	0.47 ± 0.09	2.33 ± 0.36	3.87 ± 0.75	3.85 ± 0.21	13.79 ± 5.03	10.58 ± 3.32
	2	0.54 ± 0.10	3.14 ± 0.49	4.45 ± 0.86	4.63 ± 0.25	14.27 ± 5.21	9.68 ± 3.04
	3	0.50 ± 0.09	2.52 ± 0.39	3.18 ± 0.61	2.93 ± 0.16	10.98 ± 4.01	11.68 ± 3.67
	4	0.45 ± 0.08	2.61 ± 0.41	4.21 ± 0.81	3.06 ± 0.17	12.74 ± 4.65	35.82 ± 11.24
	5	0.34 ± 0.06	1.73 ± 0.08	4.11 ± 0.32	3.72 ± 0.41	6.39 ± 0.81	10.69 ± 0.38
	6	0.59 ± 0.10	2.59 ± 0.11	1.81 ± 0.14	1.76 ± 0.19	3.14 ± 0.40	2.48 ± 0.09
	7	0.44 ± 0.08	2.53 ± 0.11	1.71 ± 0.13	2.87 ± 0.31	5.26 ± 0.66	2.90 ± 0.10
Liver fibrosis	1	0.23 ± 0.07	0.91 ± 0.10	1.32 ± 0.18	0.87 ± 0.13	3.66 ± 0.64	59.9 ± 24.39
	2	0.24 ± 0.07	1.18 ± 0.14	4.26 ± 0.57	1.76 ± 0.26	4.67 ± 0.82	74.74 ± 30.43
	3	0.15 ± 0.22	2.77 ± 0.10	10.9 ± 0.18	1.57 ± 0.19	7.97 ± 0.38	86.90 ± 0.71
	4	0.43 ± 0.13	1.21 ± 0.14	2.39 ± 0.32	1.49 ± 0.22	2.76 ± 0.49	51.86 ± 21.12
	5	0.30 ± 0.09	1.60 ± 0.18	2.04 ± 0.28	1.45 ± 0.21	3.93 ± 0.69	35.74 ± 14.56
	6	0.07 ± 0.02	2.36 ± 0.27	12.64 ± 1.71	1.99 ± 0.29	8.95 ± 1.57	72.60 ± 29.51
	7	0.14 ± 0.04	2.06 ± 0.24	8.42 ± 1.14	1.37 ± 0.20	5.37 ± 0.94	91.18 ± 37.06
	8	0.27 ± 0.08	2.44 ± 0.28	6.15 ± 0.83	1.30 ± 0.19	5.30 ± 0.93	84.82 ± 34.54
	9	0.14 ± 0.04	2.64 ± 0.30	13.23 ± 1.78	1.79 ± 0.26	5.45 ± 0.95	83.91 ± 34.17
	10	0.15 ± 0.05	2.65 ± 0.30	11.28 ± 1.52	1.70 ± 0.25	6.81 ± 1.19	54.10 ± 22.03
Liver cirrhosis	1	0.18 ± 0.04	2.97 ± 0.46	11.52 ± 2.22	6.00 ± 0.32	43.20 ± 15.76	236.76 ± 74.30
	2	0.38 ± 0.07	3.52 ± 0.55	9.09 ± 1.75	4.69 ± 0.26	39.11 ± 14.27	152.61 ± 47.89
	3	0.23 ± 0.04	5.99 ± 0.93	28.58 ± 5.50	5.18 ± 0.28	42.96 ± 15.68	417.14 ± 130.9
	4	0.34 ± 0.06	4.06 ± 0.63	16.51 ± 3.18	5.51 ± 0.30	42.56 ± 15.53	317.79 ± 99.72
	5	0.21 ± 0.04	5.32 ± 0.83	28.73 ± 5.53	3.19 ± 0.17	45.05 ± 16.43	473.96 ± 148.73
	6	0.33 ± 0.06	6.31 ± 0.99	25.51 ± 4.91	3.36 ± 0.18	44.32 ± 16.17	484.19 ± 151.94
	7	0.32 ± 0.06	5.33 ± 0.84	33.67 ± 6.48	3.00 ± 0.16	46.97 ± 17.14	424.21 ± 133.12
	8	0.42 ± 0.07	4.18 ± 0.65	17.27 ± 3.32	3.49 ± 0.19	33.34 ± 12.17	225.10 ± 70.64
	9	0.31 ± 0.11	2.93 ± 0.06	7.58 ± 0.170	2.32 ± 0.28	9.28 ± 0.41	99.46 ± 15.54
	10	0.10 ± 0.12	4.06 ± 0.05	9.89 ± 0.19	2.76 ± 0.21	14.28 ± 0.28	108.84 ± 20.72
Early-stage HCC	1	0.09 ± 0.03	2.18 ± 0.25	9.76 ± 1.32	2.44 ± 0.36	3.99 ± 0.70	83.48 ± 33.99
	2	0.05 ± 0.01	3.44 ± 0.39	10.61 ± 1.46	1.19 ± 0.18	6.87 ± 1.21	85.15 ± 35.76
	3	0.08 ± 0.02	4.65 ± 0.53	9.99 ± 1.35	1.32 ± 0.20	3.56 ± 0.62	91.18 ± 37.13
	4	0.22 ± 0.07	2.40 ± 0.27	6.62 ± 0.89	5.90 ± 0.87	2.74 ± 0.48	86.90 ± 35.39
	5	0.05 ± 0.01	2.79 ± 0.32	7.36 ± 0.99	1.37 ± 0.20	7.42 ± 1.30	89.05 ± 36.26
	6	0.04 ± 0.01	6.81 ± 0.77	25.38 ± 3.42	4.66 ± 0.69	3.17 ± 0.55	84.82 ± 34.54
	7	0.04 ± 0.01	3.22 ± 0.37	11.05 ± 1.49	3.56 ± 0.52	3.52 ± 0.62	86.90 ± 35.39
	8	0.14 ± 0.04	3.03 ± 0.34	7.77 ± 1.05	4.77 ± 0.70	3.13 ± 0.55	89.05 ± 36.26
	9	0.05 ± 0.02	3.00 ± 0.34	9.86 ± 1.33	4.37 ± 0.64	4.22 ± 0.74	87.82 ± 35.76
	10	0.13 ± 0.01	3.14 ± 0.42	13.88 ± 1.04	4.67 ± 1.37	4.08 ± 4.01	91.23 ± 22.69
Intermediate-stage HCC	1	0.27 ± 0.05	2.95 ± 0.46	13.21 ± 2.54	2.97 ± 0.16	18.76 ± 6.84	345.12 ± 108.3
	2	0.43 ± 0.08	5.52 ± 0.86	18.96 ± 3.65	8.12 ± 0.44	32.06 ± 11.69	332.10 ± 104.22
	3	0.18 ± 0.03	3.12 ± 0.49	10.82 ± 2.08	2.48 ± 0.14	26.05 ± 9.51	213.46 ± 66.99
	4	0.37 ± 0.07	2.80 ± 0.44	7.35 ± 1.42	1.83 ± 0.10	15.39 ± 5.61	120.22 ± 37.72
	5	0.17 ± 0.03	3.90 ± 0.61	15.39 ± 2.96	2.64 ± 0.15	26.21 ± 9.56	319.21 ± 100.17
	6	0.17 ± 0.03	6.08 ± 0.95	26.14 ± 5.03	3.49 ± 0.19	27.73 ± 10.11	403.86 ± 126.73
	7	0.16 ± 0.03	3.86 ± 0.60	16.27 ± 3.13	2.71 ± 0.15	42.80 ± 15.62	315.92 ± 99.13
	8	0.14 ± 0.03	8.35 ± 1.30	43.28 ± 8.33	4.69 ± 0.26	63.96 ± 23.34	799.95 ± 251.03
	9	0.07 ± 0.03	1.38 ± 0.20	12.01 ± 1.14	3.65 ± 0.48	2.35 ± 0.51	8.70 ± 0.60
	10	0.06 ± 0.02	1.48 ± 0.21	5.53 ± 0.53	3.38 ± 0.44	3.79 ± 0.84	27.77 ± 1.91
Advanced-stage HCC	1	0.01 ± 0.00	4.07 ± 0.18	16.73 ± 1.28	4.29 ± 0.47	1.73 ± 0.22	28.29 ± 1.01
	2	0.04 ± 0.01	2.16 ± 0.09	5.25 ± 0.41	1.99 ± 0.22	2.62 ± 0.33	24.45 ± 0.87
	3	0.01 ± 0.01	2.56 ± 0.11	5.97 ± 0.46	2.31 ± 0.25	1.86 ± 0.24	22.32 ± 0.80
Terminal-stage HCC	1	0.09 ± 0.03	1.82 ± 0.21	6.29 ± 0.85	1.49 ± 0.22	8.76 ± 1.53	61.42 ± 25.01
	2	0.03 ± 0.01	0.73 ± 0.08	36.42 ± 4.91	1.69 ± 0.25	9.96 ± 0.52	52.09 ± 21.21
	3	0.09 ± 0.03	1.43 ± 0.16	6.44 ± 0.87	1.36 ± 0.20	7.35 ± 1.29	63.94 ± 26.04
	4	0.11 ± 0.03	1.79 ± 0.20	5.62 ± 0.76	2.00 ± 0.29	8.60 ± 1.51	74.74 ± 30.43
	5	0.18 ± 0.06	1.71 ± 0.20	5.99 ± 0.81	2.39 ± 0.35	18.84 ± 3.30	93.18 ± 27.13
	6	0.26 ± 0.08	1.53 ± 0.18	6.39 ± 0.86	2.18 ± 0.32	23.2 ± 4.06	89.05 ± 36.26
	7	0.14 ± 0.04	2.22 ± 0.25	7.52 ± 1.01	2.19 ± 0.32	7.85 ± 1.37	90.11 ± 35.16
	8	0.15 ± 0.05	1.75 ± 0.20	7.29 ± 0.98	2.00 ± 0.29	17.29 ± 3.02	91.18 ± 37.13
	9	0.08 ± 0.02	2.19 ± 0.25	14.86 ± 2.00	1.78 ± 0.26	22.69 ± 3.97	89.05 ± 36.26

**Table 4:** Template values of expression characteristics for different liver diseases.

	Liver Disease-Prognostic mRNA Feature Template Values					
Liver Disease State	*AVPR1A*	*RCN2*	*ANXA2*	*CLEC10A*	*FABP4*	*GSTP1*
Hepatitis	0.473	2.529	3.874	3.064	10.981	10.583
Liver fibrosis	0.188	2.213	7.282	1.529	5.336	73.670
Liver cirrhosis	0.317	4.120	16.890	3.422	42.761	277.277
Early-stage HCC	0.066	3.086	9.923	3.964	3.772	87.360
Intermediate-stage HCC	0.168	3.489	14.301	3.172	26.130	317.564
Advanced-stage HCC	0.011	2.557	5.968	2.313	1.855	24.448
Terminal-stage HCC	0.105	1.751	6.445	1.996	9.965	89.005
Weight	1	1	1	1	3	5

**Table 5:** Concordance between liver diseases predicted by the “superposition analysis method” and those diagnosed by pathology.

Liver Disease State	Liver Tissue Samples	Correct Predictions	Incorrect Predictions	Prediction Accuracy
Hepatitis	7	7	0	100%
Liver fibrosis	10	9	1	90%
Liver cirrhosis	10	8	2	80%
Early-stage HCC	10	9	1	90%
Intermediate-stage HCC	10	5	5	50%
Advanced-stage HCC	3	3	0	100%
Terminal-stage HCC	9	9	0	100%

## Data Availability

The data presented in this study are available from the corresponding author upon reasonable request.

## Supplementary Materials

The following supporting information can be downloaded at: https://www.mdpi.com/article/10.3390/mps9050147/s1, Figure S1: Average ratio values of 6 prognostic mRNAs for seven liver diseases.

## Abbreviations

The following abbreviations are used in this manuscript:

HCC	Hepatocellular carcinoma
GEO	Gene Expression Omnibus
*ANXA2*	Annexin A2
*AVPR1A*	Arginine vasopressin receptor 1A
*CLEC10A*	C-type lectin domain family 10 member A
*FABP4*	Fatty acid binding protein 4
*GSTP1*	Glutathione S-transferase pi 1
*RCN2*	Reticulocalbin 2
qPCR	Real-time quantitative PCR
AFP	Alpha-fetoprotein
GO	Gene Ontology
KEGG	Kyoto Encyclopedia of Genes and Genomes
