# Supplementary material for: Superposition Analysis: A Method for Liver Disease Diagnosis and Prognostic Evaluation Related Gene Screening
Source: Methods Protoc. 2026 Oct 9;9(5):147. doi: 10.3390/mps9050147 (PMC13707675; doi:10.3390/mps9050147)
Supplement: Supplementary file 1 [file mps-09-00147-s001.zip › mps-4509114-supplementary.pdf]

Supplementary Figure

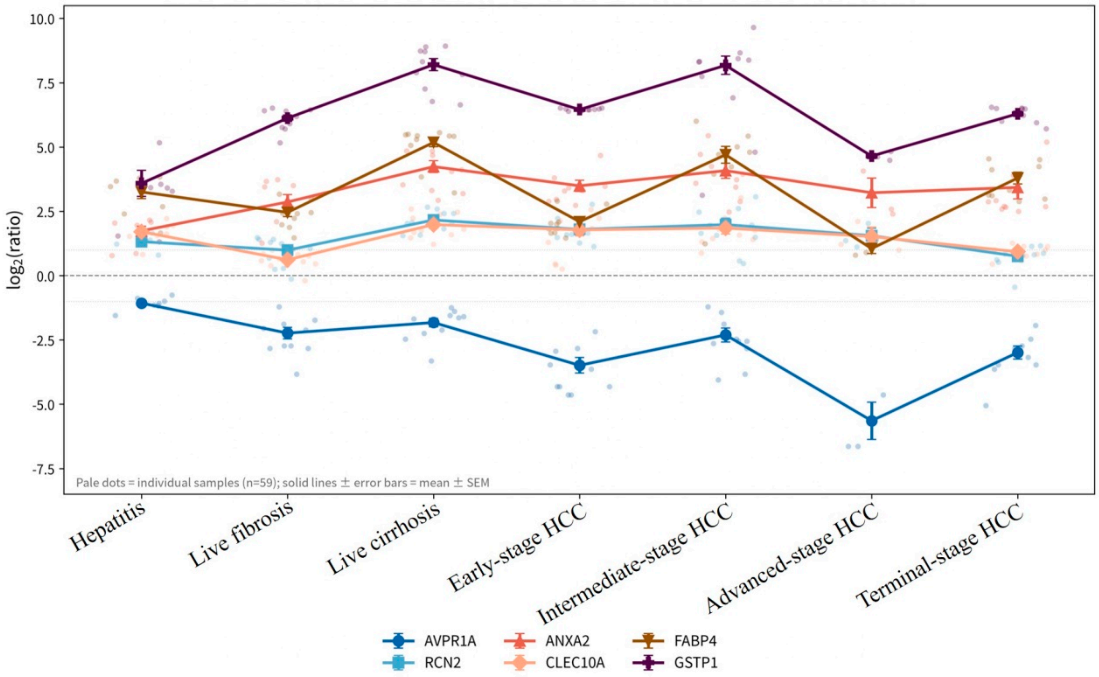

Supplementary Figure S1. Average ratio values of 6 prognostic mRNAs for seven liver diseases.
